# The Carbene Cannibal: Photoinduced Symmetry-Breaking Charge Separation in an Fe(III) *N*-Heterocyclic Carbene

**DOI:** 10.1021/jacs.1c03770

**Published:** 2021-07-15

**Authors:** Nidhi Kaul, Reiner Lomoth

**Affiliations:** Department of Chemistry—Ångström Laboratory, Uppsala University, Box 523, SE-75120 Uppsala, Sweden

## Abstract

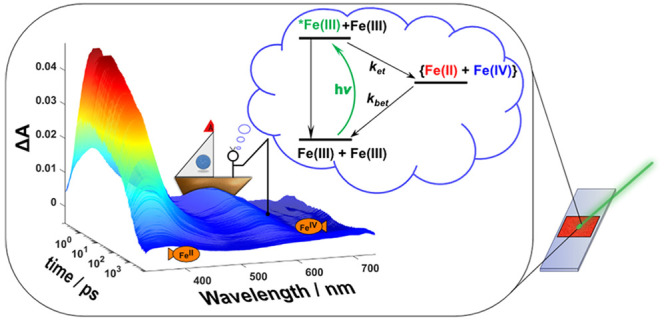

Photoinduced symmetry-breaking charge separation (SB-CS) processes offer the possibility of harvesting solar energy by electron transfer between identical molecules. Here, we present the first case of direct observation of bimolecular SB-CS in a transition metal complex, [Fe^III^L_2_](PF_6_) (L = [phenyl(tris(3-methylimidazol-1-ylidene))borate]^−^). Photoexcitation of the complex in the visible region results in the formation of a doublet ligand-to-metal charge transfer (^2^LMCT) excited state (*E*_0–0_ = 2.13 eV), which readily reacts with the doublet ground state to generate charge separated products, [Fe^II^L_2_] and [Fe^IV^L_2_]^2+^, with a measurable cage escape yield. Known spectral signatures allow for unambiguous identification of the products, whose formation and recombination are monitored with transient absorption spectroscopy. The unusual energetic landscape of [Fe^III^L_2_]^+^, as reflected in its ground and excited state reduction potentials, results in SB-CS being intrinsically exergonic (Δ*G*_CS_° ∼ −0.7 eV). This is in contrast to most systems investigated in the literature, where Δ*G*_CS_° is close to zero, and the charge transfer driven primarily by solvation effects. The study is therefore illustrative for the utilization of the rich redox chemistry accessible in transition metal complexes for the realization of SB-CS.

Photoinduced symmetry-breaking charge separation (SB-CS) processes are characterized by preferential activity of one of two or more seemingly equivalent charge separation pathways.^1−3^ Intramolecular SB-CS has received widespread attention in the literature,^4−12^ with many tailored synthetic architectures serving as models to understand design principles endemic in natural systems^13^ for efficiently harvesting solar energy. Examples of bimolecular SB-CS involving a single excited reactant (*S + S → S^+^ + S^–^) are, on the other hand, elusive, and perylene is the only documented instance^14^ with direct spectroscopic evidence characterizing the charge separated products.

General energetic considerations for SB-CS are encapsulated in eq 1:
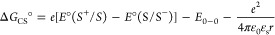
1

For typical systems studied, predominantly featuring organic chromophores, standard potentials for oxidation and reduction of the ground state (GS), *E*°(*S*^+^/*S*) and *E*°(*S*/*S*^–^), are invariably correlated with the energy of the highest occupied and lowest unoccupied molecular orbitals (i.e., HOMO and LUMO). The difference therefore corresponds to *E*_0–0_, the energy of the lowest singlet excited state as approximated by the HOMO–LUMO gap. Accordingly, the free energy change for charge separation Δ*G*_CS_° can be expected to be close to zero, where the last term in eq 1 accounts for the Coulombic work for separation over distance *r* in a medium of dielectric constant ε_s_.

Charge separation is hence typically contingent on favorable solvation effects, and factors such as the mutual spatial orientation of the chromophores and local environmental asymmetry^15−17^ (vis-à-vis the solvent) assume critical importance.

In general, SB-CS can be expected to be substantially more endergonic in transition metal complexes (TMCs) compared to organic systems, with deleterious nonradiative decay processes such as intersystem crossing or rapid deactivation to low-lying metal-centered states^18^ making it a priori energetically untenable. This is also true for recently reported TMCs where self-quenching products have been hypothesized as intermediates in photocatalytic reactions.^19−22^ Reported values indicate that the charge separation step can be expected to be fairly endergonic;^23^ hence, one may postulate that the overall process might be driven by the exothermicity of ensuing reactions. This is distinct from a case where SB-CS is intrinsically exergonic and, therefore, largely independent of solvation effects or any coupled reactions. No examples of the latter scenario exist in the literature to the best of our knowledge.

Recently, detailed photophysical characterization of a low-spin iron carbene complex,^24^ [Fe^III^L_2_](PF_6_) (L = [phenyl(tris(3-methylimidazol-1-ylidene))borate]^−^), with an open shell d^5^ electronic configuration has been reported;^25^ this complex possesses a fluorescent ^2^LMCT excited state, and its relevant properties together with the molecular structure are shown in Chart 1.

**Chart 1:**
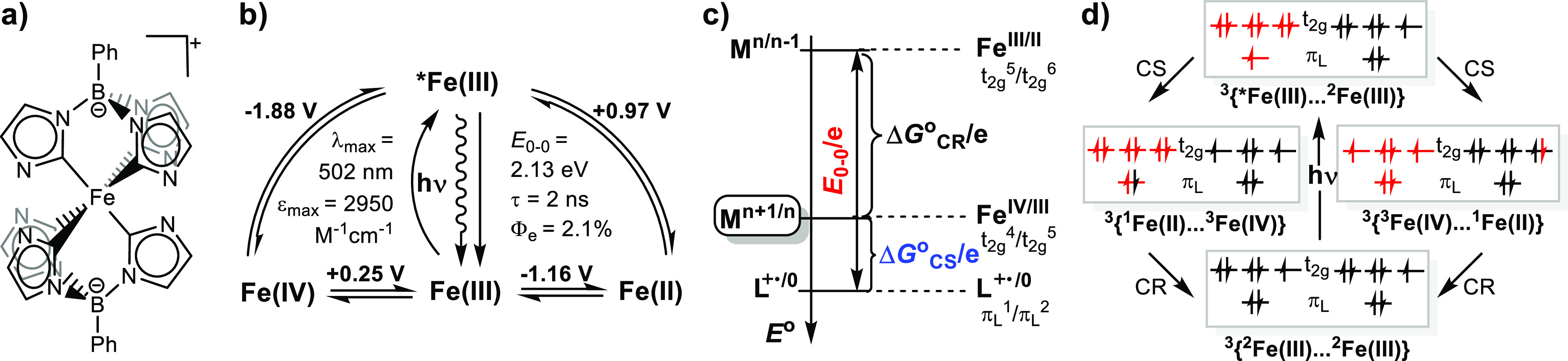
(a) Molecular Structure of [Fe^III^L_2_]^+^, (b) Latimer Diagram,a (c) Thermodynamics of SB-CS for a TMC: LMCT Excited State Energy (*E*_0-0_) and Free Energies of Charge Separation (Δ*G*_CS_°) and Recombination (Δ*G*_CR_°)b [Right: Specific Couples and Relevant Electron Configurations for the Case of an Fe(III) (d^5^ Low-Spin) Complex], and (d) Molecular Orbital Representation of SB-CSc

a All potentials referenced to Fc^+^/Fc. Δ*G*_CS_° = −0.72 eV for oxidation or reduction of the Fe(III) ground state by the ^2^LMCT excited state (*Fe(III)). b As defined by the standard potentials (*E*°) of the couples involved in the LMCT excitation (M^*n*/*n*–1^, L^+•/0^) and a second metal-centered couple (M^*n*+1/*n*^). The latter couple at intermediate potential ensures nonzero driving force for CS into M^*n*–1^ and M^*n*+1^ as long as *E*_0-0_ can be approximated by the indicated potential difference. c Indicating electron configurations of the ligand HOMO (π_L_) and the metal *t*_2*g*_ orbitals upon photoexcitation (*h*ν), charge separation (CS) by reductive (left) or oxidative (right) quenching, and charge recombination (CR).

Here, the strong σ-donation of the *N*-heterocyclic carbene ligand stabilizes higher oxidation states such that the first oxidation and reduction of the GS occurs on orbitals with predominantly metal character, at potentials below the ligand oxidation, which is implicated in the LMCT transition. In contrast to all examples in the literature, the configurations of the excited (π_L_^1^*t*_2*g*_^6^) and charge separated (*t*_2*g*_^4^, *t*_2*g*_^6^) states involve different orbitals, resulting in different energies of 2.13 and 1.41 eV, respectively (Chart 1).

Bimolecular SB-CS resulting in disproportionation into the outer metal oxidation states, [Fe^II^L_2_] (Fe(II)) and [Fe^IV^L_2_]^2+^ (Fe(IV)), can thus be expected to proceed with a notable free energy change, Δ*G*_CS_ = −0.7 eV, as calculated from eq 1. The general mechanistic considerations for the electron transfer (ET) self-quenching of *[Fe^III^L_2_]^+^ (*Fe(III)) by its GS, [Fe^III^L_2_]^+^ (Fe(III)), remain the same as that for any photoinduced bimolecular quenching reaction, described by reaction eqs 2.1–2.5.

The two reacting doublets in eq 2.1 can result in encounter complexes having singlet or triplet multiplicity. Since the formed product is a triplet, spin conservation suggests that the formation of viable encounter complexes is statistically favored three to one and can proceed with a maximal rate constant of 3/4*k*_d_. On the other hand, the diffusional encounter probability of the reactant pairs as seen in eq 2.1 can be expected to be negligible within the 2 ns ES lifetime at concentrations <5 mM. Experiments were therefore carried out at a concentration of ca. 70 mM of [Fe^III^L_2_]^+^ in acetonitrile in a 25 μm path length cell.


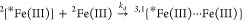
2.1


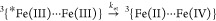
2.2


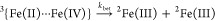
2.3


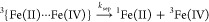
2.4


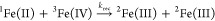
2.5

Apart from lowered emission intensity (65% quenched) as expected (Figure S2), the measured absorption, emission, and excitation spectra were unperturbed from those observed at dilutions where no self-quenching is detectable (Figure 1). Hence, no indications of behaviors commonly encountered at higher concentration regimes, such as aggregation or excimer formation, could be observed.

**Figure 1:**
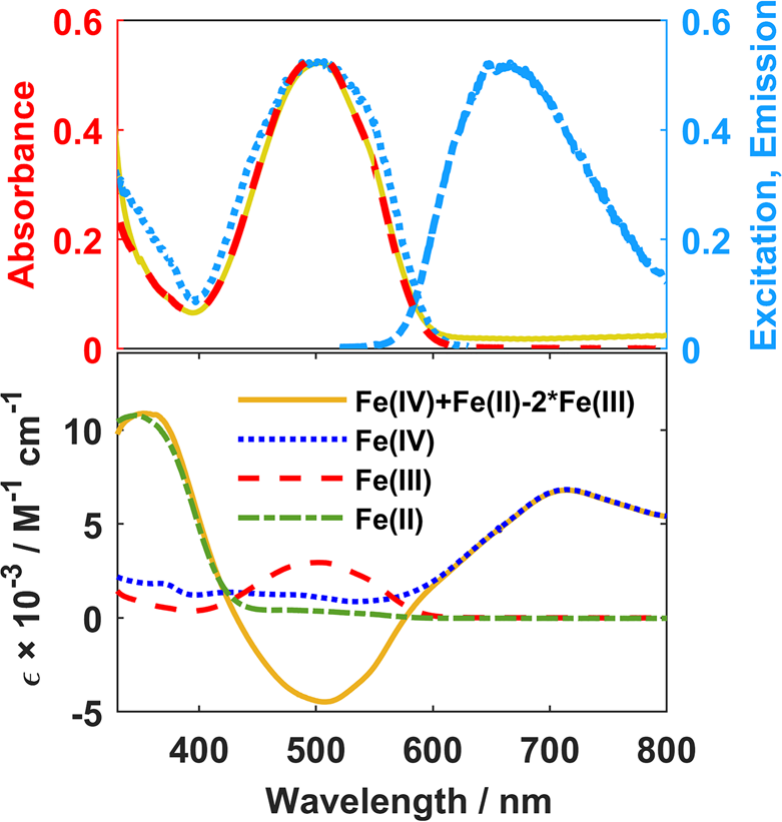
Top: Steady state data obtained for Fe(III) in acetonitrile at ca. 70 mM showing the absorption (gold), excitation (dotted blue), and emission (dashed blue) spectra, plotted together with a scaled absorption spectrum obtained at a concentration of ca. 2 mM (dashed red) where no self-quenching is detectable. Bottom: Spectral signatures of the expected charge separated products as determined from UV–vis spectroelectrochemistry.^25^

Equipped with unambiguous spectral markers for the charge separated state obtained from UV–vis spectroelectrochemistry^25^ (Figure 1), we turned to transient absorption (TA) spectroscopy on the femtosecond–nanosecond time scales to verify the occurrence of the purported SB-CS process (Figure 2).

**Figure 2:**
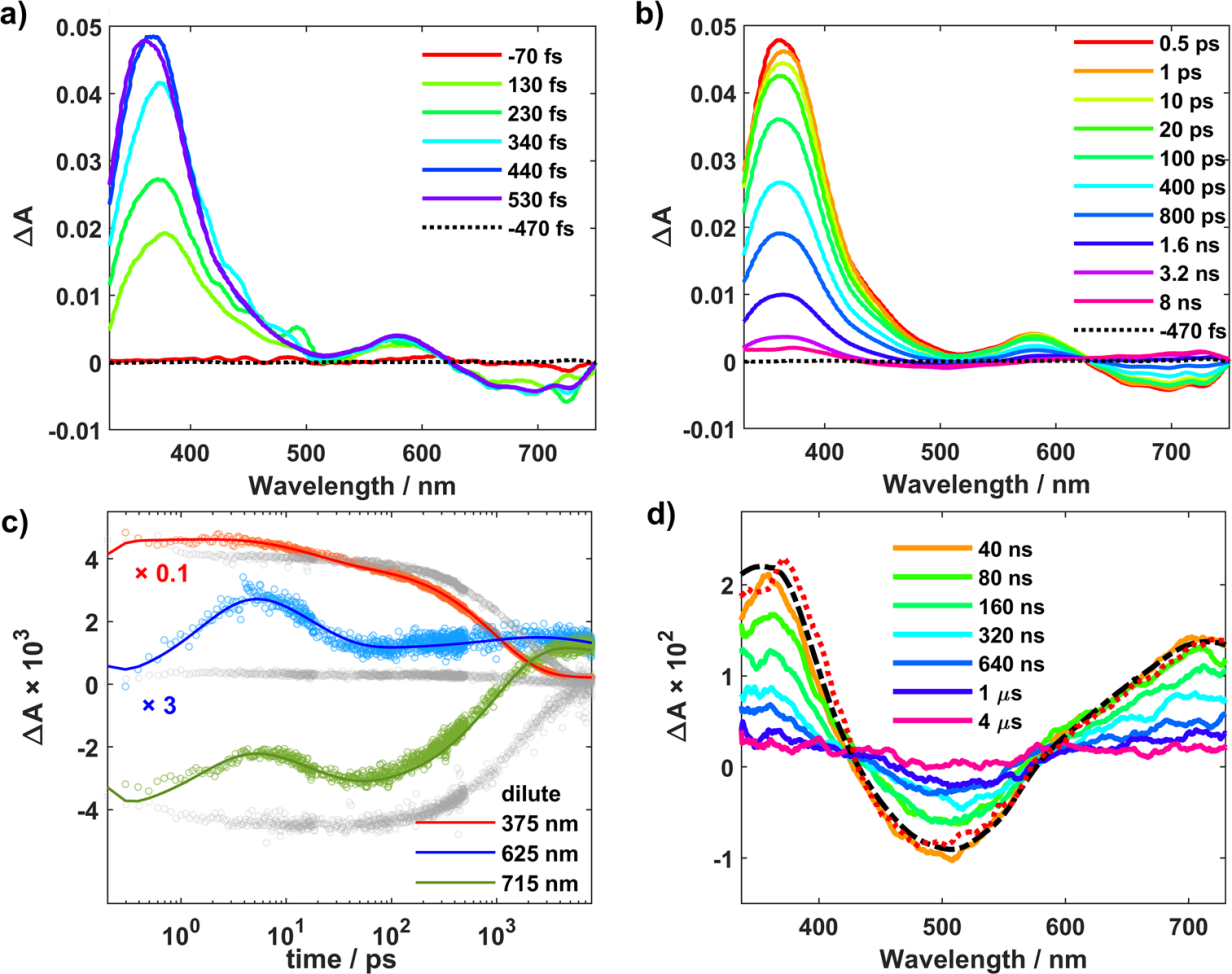
(a, b) Selected fs-TA spectra measured at early and late time scales, respectively, for a ca. 70 mM solution of Fe(III) in acetonitrile. λ_ex_ = 502 nm; power = 2 μJ/pulse. The surviving free ions can be noted in the last spectrum in part b at 8 ns (pink). (c) Kinetics measured at 375 nm (Fe(II) maximum), 625 nm (isosbestic point in the unperturbed ES decay), and 715 nm (Fe(IV) maximum). Product formation is clearly seen as a rise in the first few picoseconds at the latter two wavelengths where absorption from Fe(IV) attenuates the stimulated emission signal from the unquenched excited state. Data scaled as indicated for clarity. Data recorded at ca. 2 mM shown in gray for comparison. (d) Scaled 8 ns spectrum (dotted red) from panel b and the product spectrum (dashed black) obtained from spectroelectrochemical measurements plotted together with data from ns-TA (λ_ex_ = 465 nm, power = 15 mJ/pulse) illustrating recombination of the CS products.

At the concentration employed, approximately 30% (calculated from Poisson statistics, and in good agreement with the observed steady state emission quenching, see SI) of the reactant pairs have a probability of being at near contact distances upon photoexcitation. These contact pairs (CPs), i.e., *[Fe^III^L_2_]^+^ and [Fe^III^L_2_]^+^ molecules already having the appropriate mutual distance and orientation to undergo ET, can be expected to react first. Accordingly, product formation is clearly seen in the first few picoseconds as a rise in the kinetics in Figure 2c observed at 625 nm (cf. dilute ES decay, Figure S4, where it is an isosbestic point) and 715 nm, due to pronounced absorption from the formed [Fe^IV^L_2_]^2+^, which has a broad band in the red. In the spectra, Figure 2a and Figure S5, formation of [Fe^IV^L_2_]^2+^ causes attenuation of the stimulated emission band intensity, while that of [Fe^II^L_2_] blue-shifts the ^2^LMCT absorption peak from ∼375 to 360 nm. In Figure 2b, the spectral red-shift to ∼365 nm seen from 500 fs to 1 ps can be tentatively attributed to changes in the solvation environment to accommodate the charge separated state, where the neutral [Fe^II^L_2_] is now present with the doubly charged [Fe^IV^L_2_]^2+^. The formed products build to a maximal concentration in around 4 ps, and this is followed by ultrafast recombination in these geminate pairs on the time scale of a few tens of picoseconds, which is accompanied by a small but discernible blue-shift back to ca. 360 nm. A monotonic decrease in the spectral intensity is observed thereafter, which eventually reveals the products that escape geminate recombination, after the decay signal from the unquenched proportion of the excited state disappears (>2 ns); the bleach expected at around 500 nm, previously obscured by the ES signal, also becomes evident at these later time scales (see SI for relevant decay associated spectra). The long-lived free ions, formed in equimolar amounts with a total product yield φ_CS_ ≈ 4% determined from scaled actinometry (SI), undergo recombination on the microsecond time scale to quantitatively return [Fe^III^L_2_]^+^ (Figure 2d). No signs of photodegradation were found, as adjudged from the unchanged absorption spectra before and after measurements; further, no notable power or wavelength dependence was observed (SI).

Situations where reactant distance distributions are inhomogeneous are accurately treated using coupled reaction-diffusion equations which account for the time and distance dependent “rate constant” by means of introducing a pair distribution function,^26^ and we direct the interested reader to several notable studies which target such problems in significant detail.^27−32^ In this case, since the solvent used is polar and of low viscosity, with a quencher concentration of <0.1 M, we used a sum of exponentials to perform the global fit (see SI for details), using a formal kinetic scheme, where the ultrafast events are still treated as separate steps, not coupled by diffusion. With the added flexibility of taking the diffusive reactant pairs and those at contact as two limiting cases, in a sequential model, charge separation in the latter is modeled as the first exponent, while the second exponent accounts for recombination in the geminate pairs that competes with escape in parallel. The third exponent gives the reduced fluorescence lifetime and, consequently, yields the pseudo-first-order ET rate constant for the diffusional pairs. This first analysis allows for an adequate qualitative description of the system, summarized in Scheme 1.

**Scheme 1:**
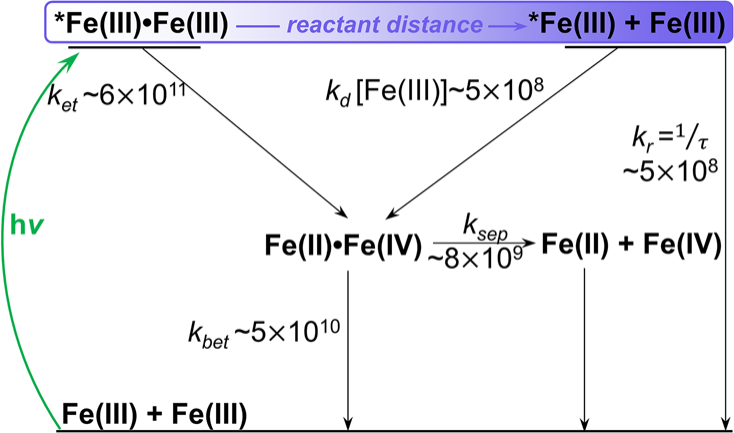
Kinetic Parameters of the SB-CS Process Obtained from Global Analysis of the Data Using a Sum of Exponentials Fit Model All rate constants in s^–1^.

Owing to the near contact distances, the electronic coupling in the CPs can be assumed to be larger than in reactant pairs that are further apart; CS is therefore ultrafast, with *k*_et_ ∼ 6 × 10^11^ s^–1^. With *k*_sep_ calculated as ∼8 × 10^9^ s^–1^ from the Debye–Smoluchowski equation, *k*_bet_ ∼ 5.2 × 10^10^ s^–1^ can be estimated from the second fit component of ∼17.8 ps. Therefore, around 15% of the geminate pairs can be expected to escape the cage, relatively higher than that typically reported for contact ion pairs.^33−35^ Structural differences in this system and those previously studied, with the latter being linked D–A systems of planar organic molecules, e.g., TCNE-pyrene, taken together with the fact that [Fe^II^L_2_] is neutral, and the formed CS products thus do not experience Coulombic attraction, could result in the higher observed survival probability.

For the diffusional pairs, the pseudo-first-order rate constant determined from the global fit, 5.4 × 10^8^ s^–1^, agrees well with that evaluated from TCSPC (4.4 × 10^8^ s^–1^, Figure S3), corresponding to a near diffusion-controlled rate constant of 7.7 × 10^9^ M^–1^ s^–1^. With a quenching efficiency of 0.65, the recombination rate determined from the overall φ_CS_ is ∼7.5 × 10^10^ s^–1^ (φ_CS_ = φ_*q*_φ_sep_; from eqs 2.3 and 2.4, ), which can be considered an “average” for all the geminate pair subpopulations, and agrees fairly well with the *k*_bet_ observed for CPs, especially considering the fact that the ultrafast time constants have larger uncertainty because of the multiexponential fit model employed.

Assuming CR to the ground state, the driving force for recombination is ∼1.4 eV, and if the internal reorganization is thought to be minimal, as suggested by negligible observed structural differences between [Fe^III^L_2_]^+^ and [Fe^IV^L_2_]^2+^,^36^ CR could take place in the inverted region,^37^ resulting in an observable yield despite the spin allowed nature of the back-electron transfer. Regardless, the prognosis is positive for the design and optimization of such systems to improve performance. For instance, by modifying the ligands so as to tune the reduction potentials, there is room for reducing the driving force for CS without compromising *k*_et_, since it can be expected to be maximal already at lower exergonicities. This would result in CR occurring deeper into the inverted region, potentially improving yields. Further, *k*_bet_ can be expected to maximize at distances larger than contact,^38−43^ unlike the normal region, potentially suppressing recombination in contact pairs.

In summary, we have demonstrated clear evidence of SB-CS in an earth abundant TMC, where the process is intrinsically exergonic and part of the CS products are long-lived. Apart from the possibility of driving chemical reactions in solutions, this study is also suggestive for the use of such complexes to drive charge separation in the solid state, allowing for fabrication of, e.g., solar cells by harvesting the CS state. We have obtained the first evidence for charge separation in thin films in our lab, and further investigations are currently underway.

## Supporting Information Available

Experimental details, steady state spectroscopy, TCSPC, additional fs-TA data, actinometry, fitting procedures, and general thermodynamic considerations (PDF)
