# Supplementary material for: The Carbene Cannibal: Photoinduced Symmetry-Breaking Charge Separation in an Fe(III) N-Heterocyclic Carbene
Source: J Am Chem Soc. 2021 Jul 15;143(29):10816–21. doi: 10.1021/jacs.1c03770 (PMC8397313; doi:10.1021/jacs.1c03770)
Supplement: Supplementary file 1 — ja1c03770_si_001.pdf [file ja1c03770_si_001.pdf]

Supporting Information:

The Carbene Cannibal: Photoinduced Symmetry-Breaking Charge Separation in an Fe(III) N-Heterocyclic Carbene

Nidhi Kaul, Reiner Lomoth*

Department of Chemistry − Ångström Laboratory, Uppsala University
Box 523, SE-75120, Uppsala, Sweden

*Corresponding Author:

Reiner Lomoth (reiner.lomoth@kemi.uu.se)

Contents

1.	**Experimental**	S3
2.	**Steady State Spectroscopy**	S5
3.	**Time Correlated Single Photon Counting**	S7
4.	**fs Transient Absorption Spectroscopy**	S8
5.	**Fitting Procedures**	S14
6.	**ns Transient Absorption Spectroscopy**	S16
7.	**General Thermodynamic Considerations**	S17
8.	**References**	S18

1. Experimental

Chemicals. Acetonitrile (for spectroscopy Uvasol®, purity ≥ 99.9 %) was obtained from Sigma Aldrich (now Merck) and used as received.

Sample Preparation. The cell used for the measurements was constructed by using a microscope slide and coverslip (18x18 mm), with a 25 μm polymer spacer (Meltonix 1170-25, Solaronix) in between the two, cut to appropriate dimensions, which could then be sealed with the help of a solder at ca. 100°C. Two horizontal slits were left as gaps in the spacer, so as to allow for pipetting of the sample – $[Fe^{III}L_2]PF_6$ dissolved in acetonitrile – via capillary action. The openings were then immediately sealed with the help of a two-component sealing mixture (Amosil 4, Solaronix) to prevent evaporation. All samples were sealed under air, in ambient conditions.

Steady-State Measurements. Absorption measurements were carried out on Varian Cary 50 and 5000 spectrophotometers. Steady state excitation and emission measurements were recorded on a Horiba Jobin Yvon Fluorolog using a front face detector geometry (60° angle), and corrections made for fluctuations in the light source and the detector response. A slit width corresponding to a spectral resolution of 5 nm was used, and the integration time was 1 s.

Time-Resolved Measurements. ns-transient absorption measurements were carried out using a Q-switched Nd:YAG laser (Model NT342B, EKSPLA; FWHM = *8* ns), whose fundamental emission at *1064* nm was frequency tripled to produce 355 nm light, which was then used to pump an optical parametric oscillator (OPO) equipped with type II nonlinear BBO crystals, so as to generate the desired pump wavelength in the visible (465 or 500 nm). The laser repetition rate was *10* Hz, data acquisition was carried out at 1 Hz, and the pump energy was maintained at *15* mJ/pulse (±*10*%). The setup utilized a near 60° detection geometry between pump and probe, and the (white) probe light came from a Xe arc lamp operated in pulsed mode for improved signal-to-noise ratio. Timed shutters prevented prolonged light exposure to the sample.

The LP920 detection system (Edinburgh Instruments) equipped with a photomultiplier tube and an Andor iStar CCD camera was used to acquire kinetic and spectral data, which was collected using the L900 software (which controlled the Tektronix digital oscilloscope as well as the CCD camera) on the connected computer. The decay traces were recorded with a consistent resolution of *5* nm, and for spectral measurements the monochromator was positioned at *575* nm. Further, the gate width was typically kept at around *10%* of the gate delay, and raw counts were kept at around *50,000*. Every measurement was averaged over *10* shots.

fs-transient absorption measurements in the visible were carried out on a setup previously described;[1,2] briefly: the output of a Coherent Libra Ti:Sapphire amplifier (795 nm, 3 kHz, 1.5 mJ, fwhm ~40 fs) with integrated oscillator and pump lasers was split into a pump and probe beam. The excitation wavelength (~502 nm) was generated by directing the pump beam into the optical parametric amplifiers (TOPAS-C, Light Conversion), while the fundamental of the amplifier was focused on a CaF_2 or Sapphire crystal (Newport TAS), in order to generate the white light supercontinuum in the ranges of ca. 330 nm to 740 nm or 490 nm to 750 nm, respectively. The probe spectrum was detected using a custom-made silicon diode array from

Newport. Pump-probe overlap was optimized at the sample, and the pump power was typically adjusted to 3 mW (standard deviation < 5%, unless otherwise noted), except for the power dependence measurements, where it was varied from 1 mW to 5 mW. A mechanical optical delay stage was used to collect data at different timepoints by varying the delay of the probe with respect to the pump, and a range of −5 ps to 8 ns was scanned. Five scans were averaged for each measurement.

Time-correlated single photon counting (TCSPC) measurements were carried out on a home-built setup previously described[3], which employed a picosecond diode laser (Edinburgh Instruments, EPL470) at 470.4 nm (ca. 87.3 ps pulses) as excitation source, and an MCP-PMT (R3809U-51, Hammamatsu) cooled to around -40°C as the detector. The electrical trigger signals from the laser and from the detector were passed through discriminators (Tennelec TC454 and Ortec 9307, respectively) before being directed to the time to amplitude converter (TAC; Ortec 566 with 50 ns range). The TAC output was read with the help of a Data Acquisition Card (Trump PCI) with a multichannel analyzer (MCA) having 4096 channels, and collected with a Horiba Jobin Yvon DataStation 2.3. A blank microscope slide was used to scatter a portion of the exciting laser light into the detector in order to obtain the instrument response function. All measurements were carried out in reverse mode using magic angle polarization, and a cutoff filter was used to block out the excitation light. The pulse intensity was attenuated to ensure the detected photon counts were kept below 1% of the excitation frequency (20 MHz).

2. Steady State Spectroscopy

4.1 Overview and Sample Concentration

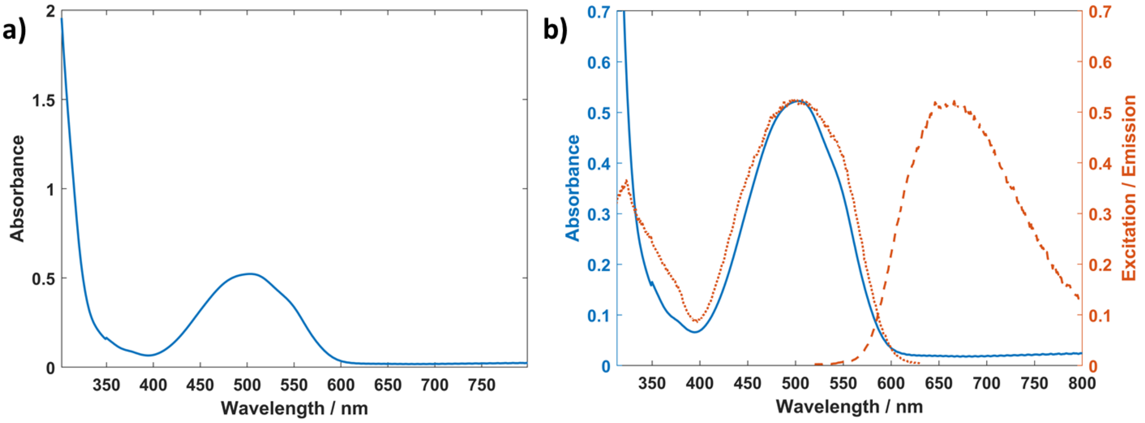

Figure S1: a) Steady state absorption spectrum of $[Fe^{III}L_2]^+$ in acetonitrile; the spectrum is unperturbed from that observed at conventional dilutions, and allows for the determination of the concentration using Beer's law ($A = \varepsilon c l$; $\varepsilon_{502} = 2950\ M^{-1} cm^{-1}$, $l = 25\ \mu m$, $A = 0.5$), which is ca. 68 mM. b) Excitation spectrum (dotted orange line; detection monochromator fixed at emission maximum of 650 nm) of the same sample overlayed on the recorded absorption spectrum, clearly showing the observed emission is from the lowest energy LMCT band. The emission maximum and bandshape (dashed orange line) is unchanged from that observed previously at typical concentrations for fluorescence measurements (ca. 20 µM). Excitation wavelength, $\lambda_{ex} = 502\ nm$.

4.2 Steady State Emission Quenching

Experimentally, to a first approximation, the extent of self-quenching can be estimated by scaling the emission intensity of a dilute sample (in an identical cell, under identical conditions, where negligible self-quenching can be expected) by a factor (10^{-Abs}) accounting for the difference in absorbed photons by means of Beer's law. In an ideal scenario, this extrapolation should result in the expected emission intensity of a high concentration sample in the absence of any self-quenching. The relevant absorption and emission data is presented in Table S1 and Figure S2, together with the results obtained from the scaling. Three excitation wavelengths, 500, 475, and 465 nm were used to check the method for consistency. Deviations are noted in the so determined factors, up to ~22%, and this resulted in the percentage quenching determined from exciting at different wavelengths to vary from 63 to 69%.

Table S1: Steady State Absorption and Emission Data

	**8 mM**			**68 mM**				
**λ/nm**	*Abs*	$1\text{-}10^{-Abs}$	Em_8	Abs	$1\text{-}10^{-Abs}$	Em_{68}	$Em_{68,\ nq}$ [a]	$Em_{68}/Em_{68,nq}$
**500**	0.06	0.129	0.38	0.5	0.684	0.74	2.01	0.37
**475**	0.05	0.109	0.33	0.45	0.645	0.67	1.96	0.34
**465**	0.04	0.088	0.31	0.4	0.602	0.63	2.09	0.30

[a]Expected unquenched intensity at 68 mM: $Em_{68,\ nq} = Em_8\{(1\text{-}10^{-Abs})_{68}/(1\text{-}10^{-Abs})_8\}$

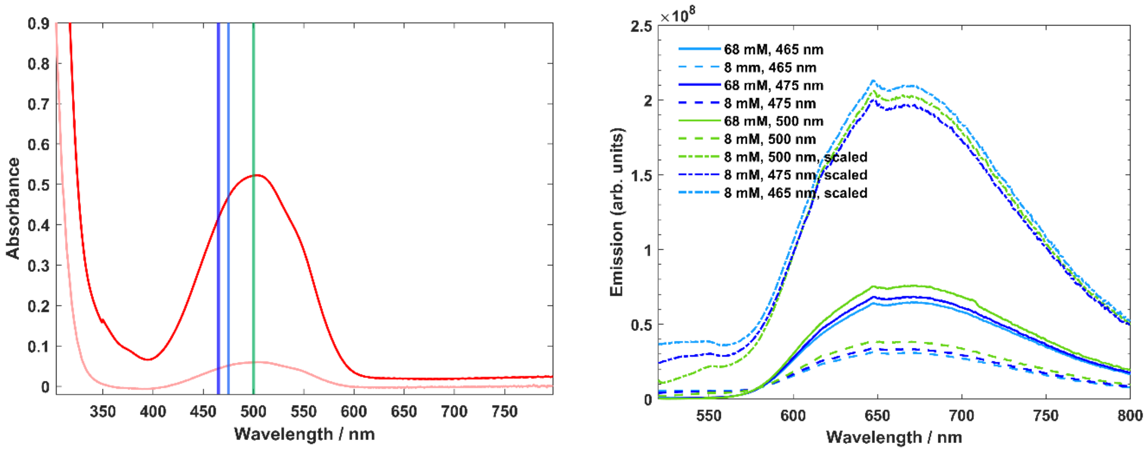

Figure S2: Steady state absorption data obtained for samples at 8 mM (light red) and 68 mM (dark red), respectively (left), plotted together with the excitation wavelengths marked with vertical lines. Steady state emission data (right). Solid lines: 68 mM; dashed lines: 8 mM; dotted lines: 8 mM data scaled for difference in absorbance. Excitation wavelengths: 500 nm (green), 475 nm (blue), 465 nm (turquoise).

Crystallographic data suggests a first approximation of iron carbene's radius as ca. 7 Å. In a sphere of action model[4] where adjacent quenchers can be thought to quench the excited state immediately, therefore can be modelled as an essentially "static" contribution i.e. being dark for a sufficiently long IRF[5,6] (these pairs would still be observable when using a fs time resolution). The sphere of action is typically assumed as the sum of the radii of the fluorophore and quencher. Assuming similar radii for the ground and excited state of $[Fe^{III}L_2]^+$ yields a sphere of volume, $V \approx 1.149 \times 10^{-20}$ cm^3. The probability of at least one molecule being in the sphere can be calculated using Poisson statistics, $P(n) = \frac{\lambda^n}{n!}e^{-\lambda}$, with n=1 and $\lambda = \frac{VNc}{1000}$, where V is the volume of the sphere, N is Avogadro's number, and c is the concentration in M, which in this case is ~0.07. This yields a probability of ca. 30%, which is similar to what is observed experimentally, when accounted for together with the diffusional component that harvests about half of the remaining 70% of the excited state population (see next section). This model and first approximation, of course, does not account for any interaction(s) between the molecules – therefore the relatively good agreement can be thought to reflect the lack of significant interactions between the molecules, likely owing to their unit positive charge and polar solvent.

3. Time-correlated Single Photon Counting (TCSPC)

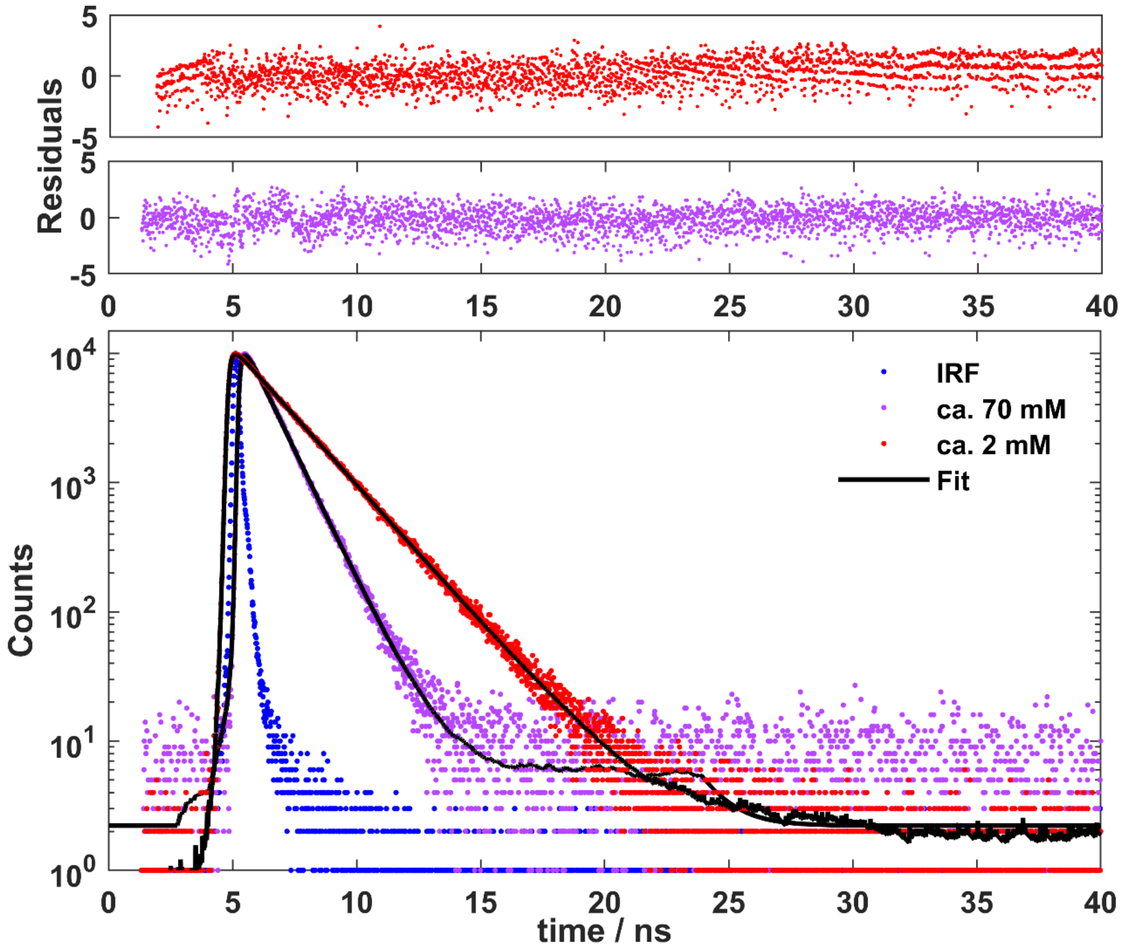

Figure S3: Time-correlated single photon counting data corrected for background plotted together with the IRF (blue); red: 2 mM sample, purple: 70 mM sample. A monoexponential fit yields a time constant of ~2 ns for the dilute sample, while it is 1.06 ns for the concentrated sample, yielding a pseudo first order dynamic quenching rate constant of ~4.4×10^8 s^{-1}. The residuals indicate an observable transient effect in the beginning; the sinusoidal nature is indicative of non-exponential processes in the system (increasing the number of exponential terms only marginally improved the fit quality).

4. fs-Transient Absorption Spectroscopy

This section compiles additional fs-data recorded for bimolecular symmetry breaking charge separation of $[Fe^{III}L_2]^+$ in acetonitrile when using different powers, crystals (CaF_2 and Sapphire) to generate the white light continuum, and a different excitation wavelength (532 nm), and some comparisons thereof. Excitation wavelength made no substantial difference to the observed dynamics, and the power dependence was found to be linear. The excited state decay observed in a dilute sample (ca. 2 mM) is also shown. Details on scaled actinometry using $[Ru(bpy)_3]^+$ as an actinometer can be found towards the end of the section. Spectra were smoothed in MATLAB using a moving average filter before plotting.

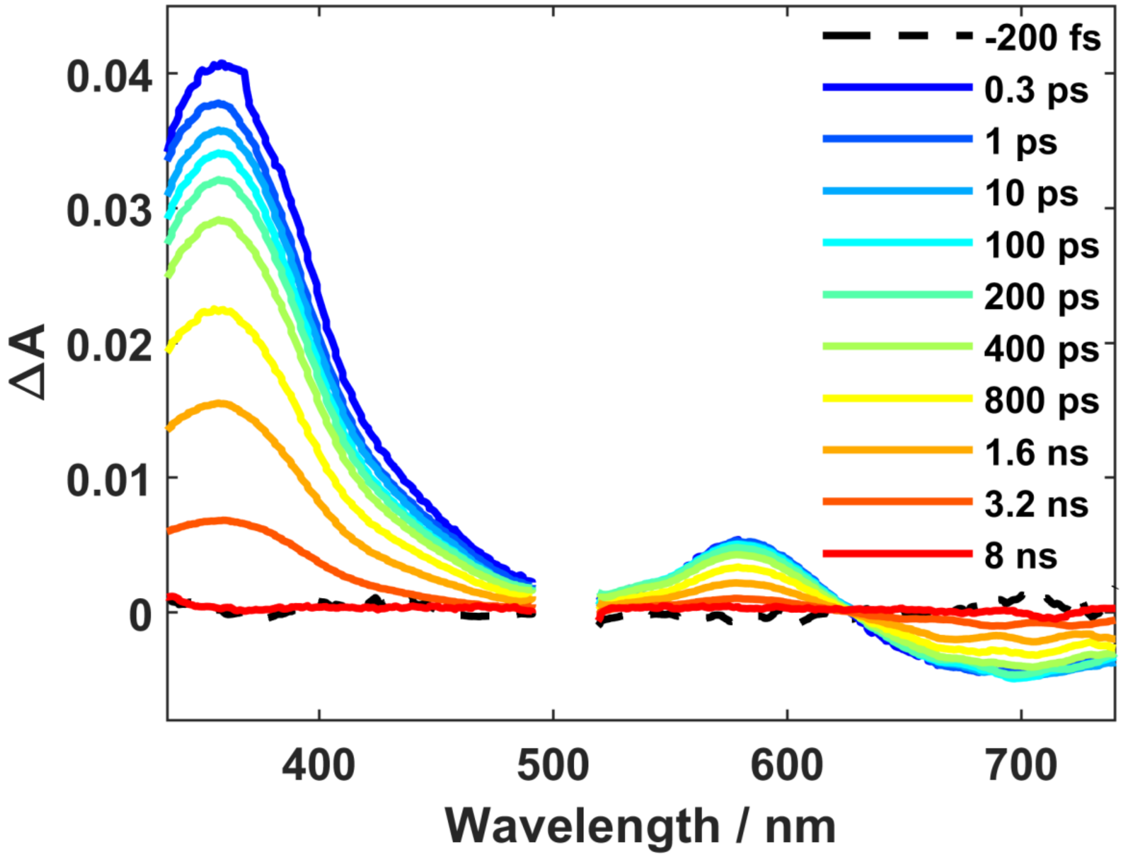

Figure S4: fs-TA spectra recorded using the CaF_2 crystal ($\lambda_{ex} = 502\,nm$, power = 3 mW) for a dilute concentration sample of 1.7 mM (absorbance = 0.5 at excitation wavelength, pathlength = 1 mm). Note the isosbestic point at ca. 625 nm. The excited state absorption peak in the blue is at 358 nm.

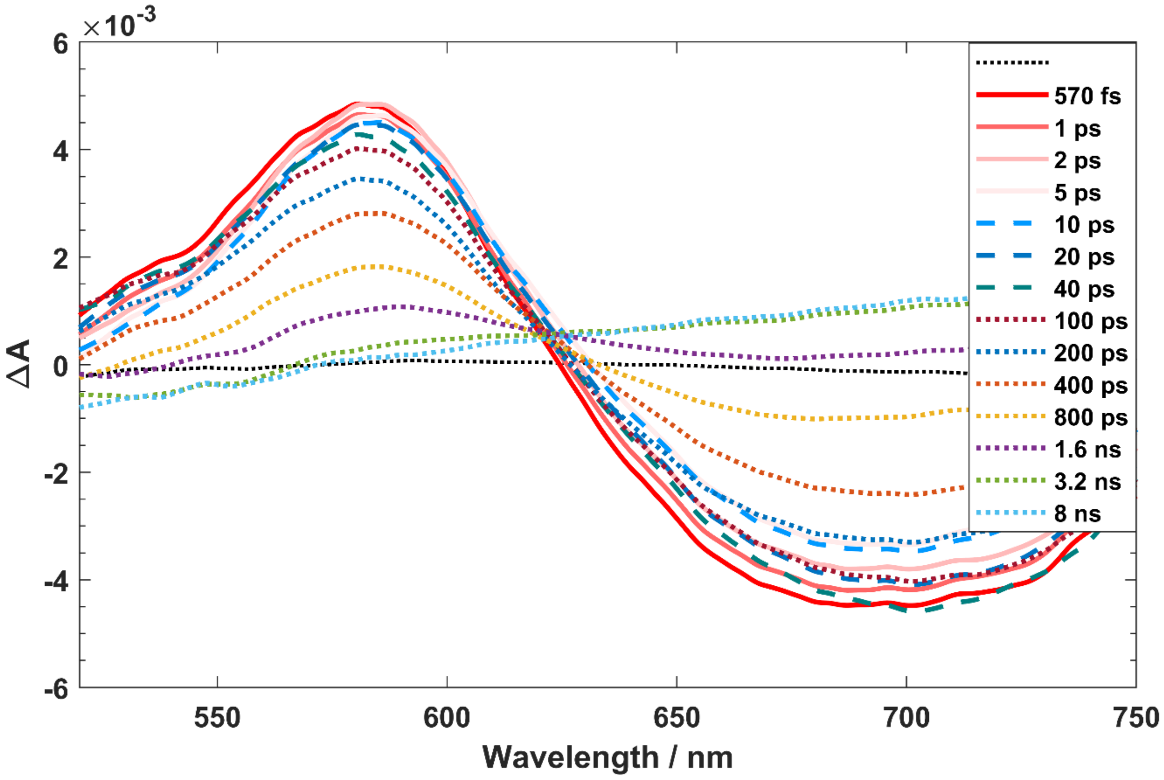

Figure S5: fs-TA spectra recorded for the same sample as that seen in Fig. 2 a) and b) in the main text, using the Sapphire crystal ($\lambda_{ex} = 502\,nm$, power = 3 mW), to better see the spectral changes in the red.

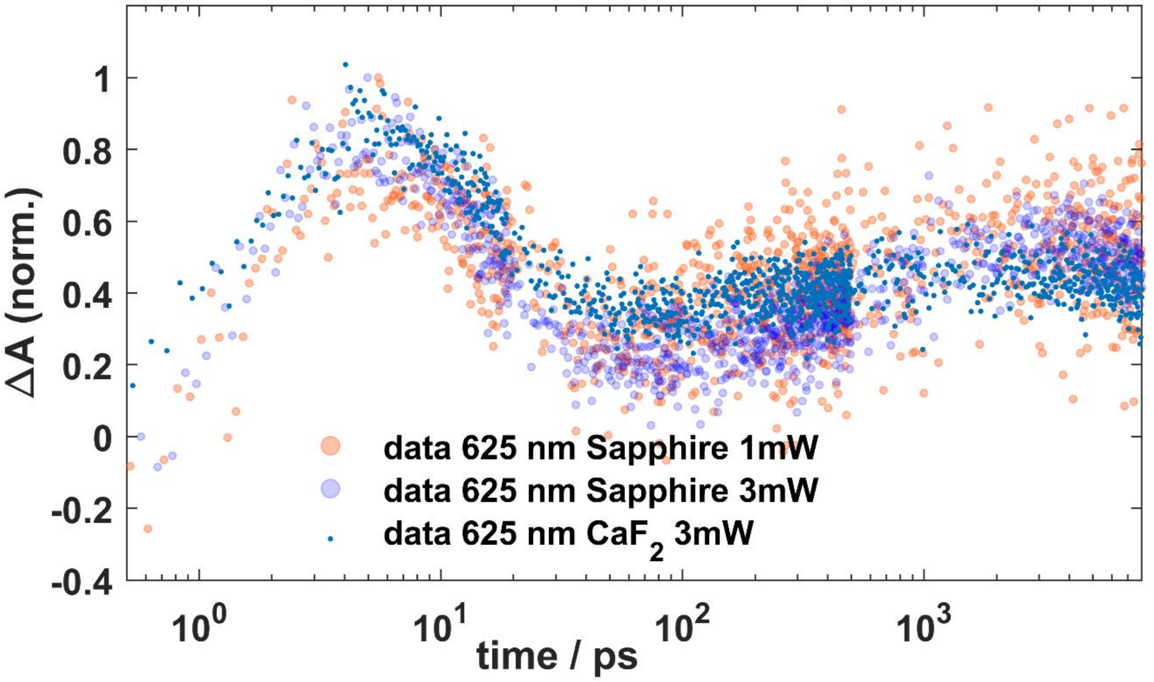

Figure S6: Comparison of kinetic traces at 625 nm recorded at different powers, and using different crystals to generate the white light continuum, as noted in the legend. Data has been normalized.

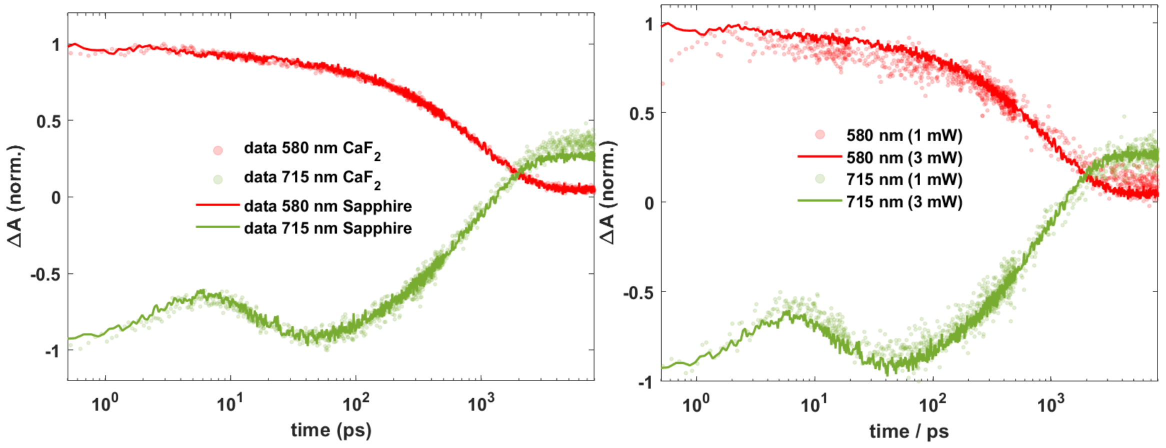

Figure S7: Comparison of kinetic traces at 580 and 715 nm recorded at different powers (Sapphire crystal, right), and using different crystals to generate the white light continuum (comparison at 3 mW power, left). Data has been normalized.

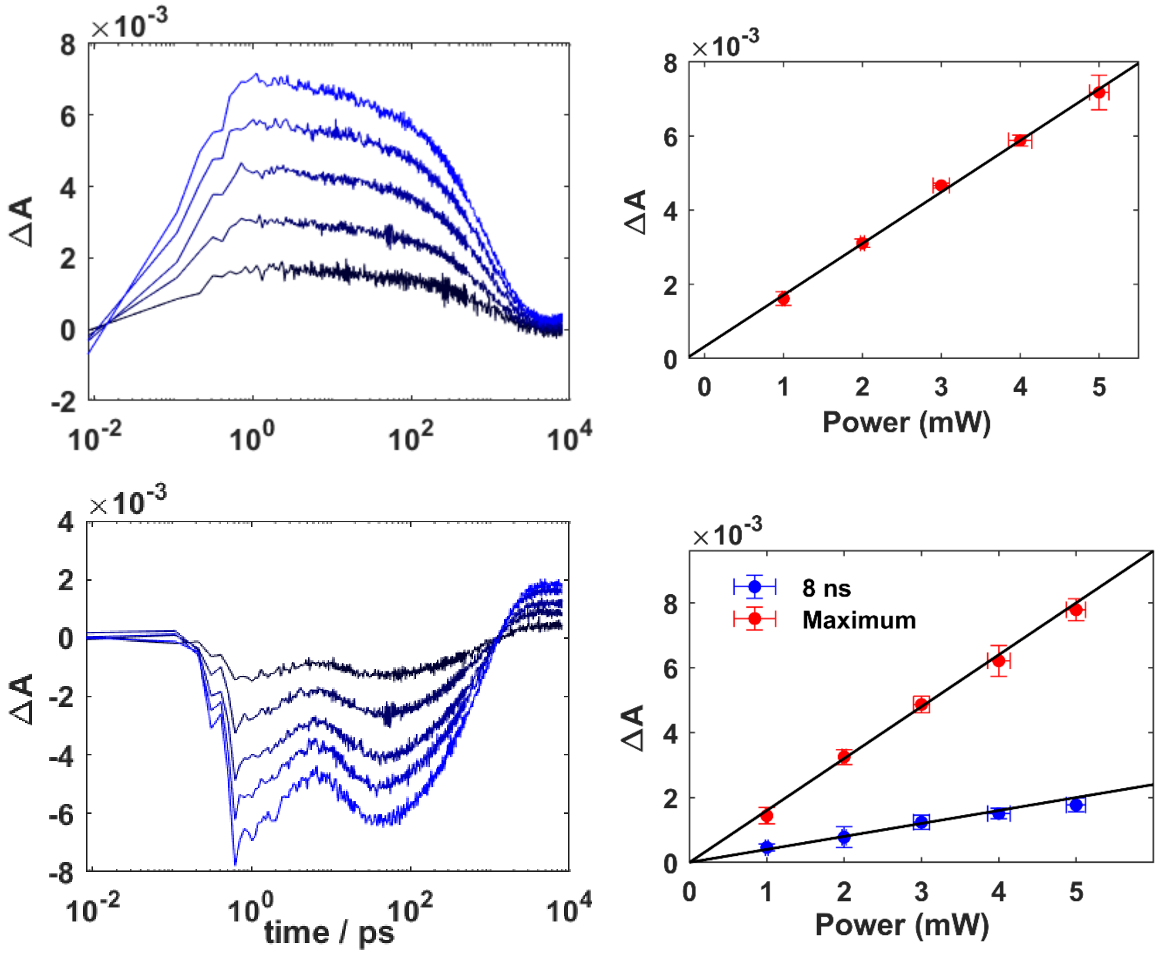

Figure S8: Linear dependence of the signal intensity on power. Top panel: 580 nm; bottom panel: 715 nm (red: signal maximum; blue: signal at 8 ns).

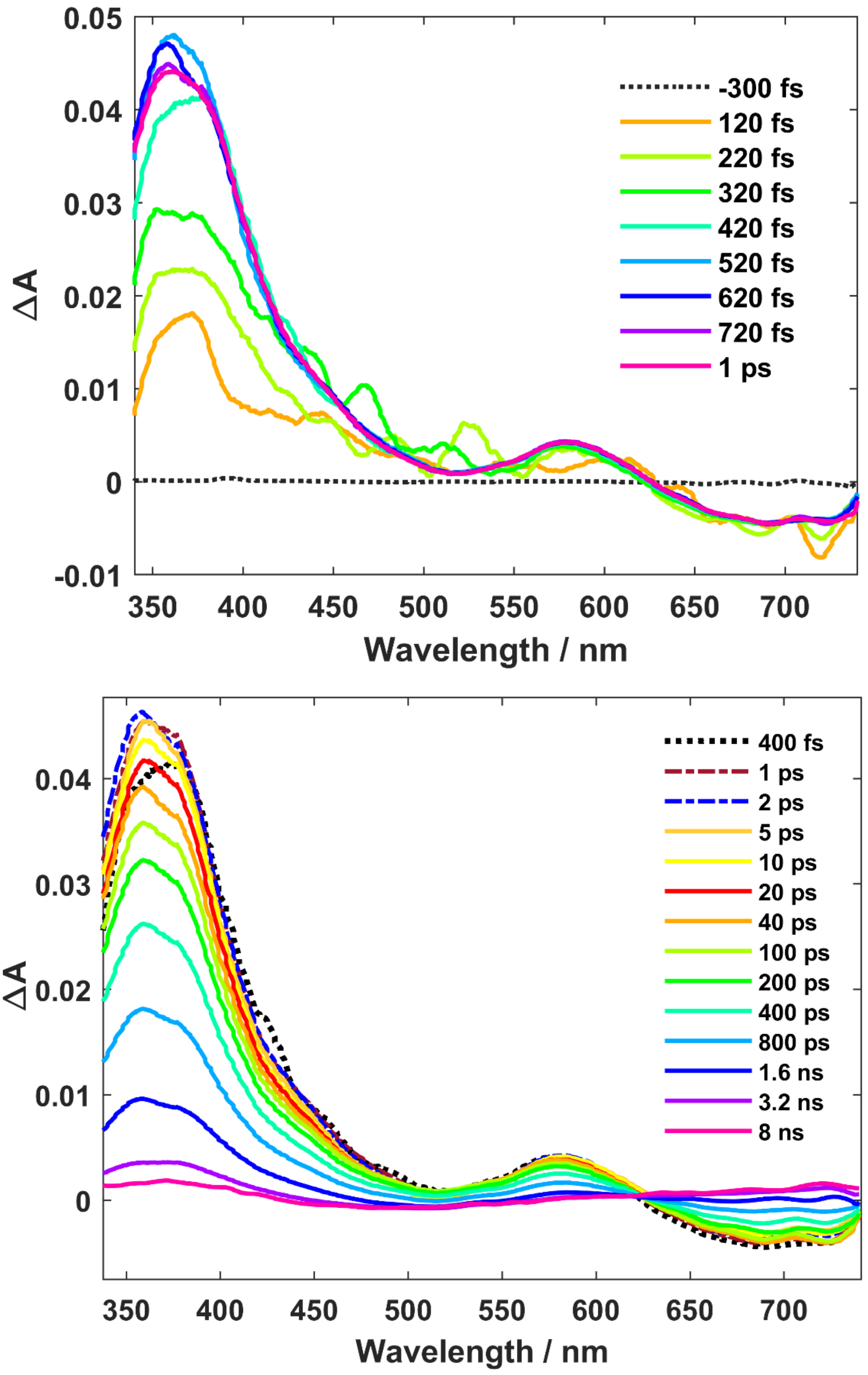

Figure S9: fs-TA spectra recorded for the same sample as that seen in Fig. 2 a) and b) in the main text, using the CaF_2 crystal but with $\lambda_{ex} = 532\ nm$, power ca. 3 mW, absorbance ca. 0.4 at the excitation wavelength. Top: early times; bottom: later times.

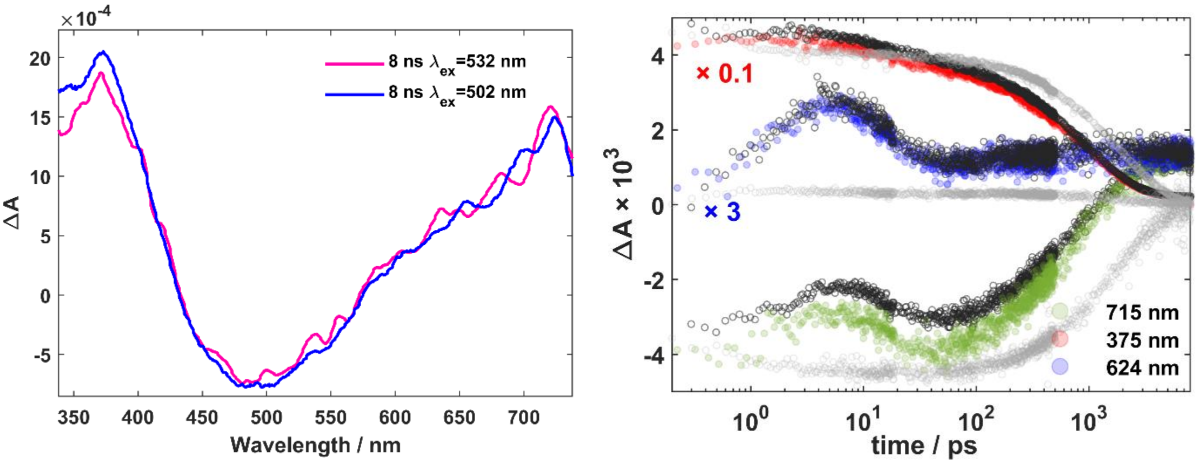

Figure S10: *Left:* Comparison of fs-TA spectra obtained at 8 ns, showing the charge separated products, recorded when exciting the sample at 502 and 532 nm. *Right:* Comparison of kinetics obtained when exciting at 532 nm (375 nm, red; 624 nm, blue; 715 nm, green) with that obtained when exciting at 502 nm (black circles). Grey circles is data recorded at the same wavelengths, but for the dilute sample shown in Fig. S4.

Product Yield Determination

In the crudest first estimate, where the differential extinction coefficients of the charge separated Fe(II) and that of the excited state in the blue (which is also an Fe(II), since it is an LMCT transition) can be assumed to be similar, the amplitudes at 375 nm suggest a total yield (0.002/0.045) of around 5%.

For more accuracy, chemical actinometry can be employed. Insofar as the initial excited state concentration can be determined with a reference dye excited under identical conditions, one can evaluate the yield of the products from the known extinction coefficients for Fe(II) and Fe(IV) previously calculated from spectroelectrochemical measurements[7] (reproduced in the bottom panel of Fig. 1 in the main text). The first condition proved difficult to achieve: a number of reference dyes, whose differential extinction coefficients are well-known, if dissolved in high enough concentrations to achieve the same absorbance as iron carbene in the same path length cell either aggregated, or could not be dissolved; sometimes the power used suggested a possibility of two photon processes and/or degradation (iron carbene's extinction coefficient is relatively modest, at around 2950 M^{-1} cm^{-1} at the maximum, allowing for use of higher powers without deviation from linearity).

$[Ru(bpy)_3]^{2+}$ was therefore used as an actinometer, whose differential extinction coefficients have recently been determined to good accuracy, and has been suggested as a robust actinometer for transient absorption spectroscopy[8]. The reference solution was made in a 1 mm (i.e. 1000 µm) path length cell, such that the absorbance at the excitation wavelength was the same as the 25 µm cell(s) used for the SB-CS experiments, and excited under identical conditions. If the only change introduced by the larger path length is a bigger excitation volume, everything else remaining the same, Beer's law suggests the initial excited stated concentration evaluated via these means can simply be scaled by a factor of 1000/25 i.e. 40 to obtain the initial excited state concentration in the 25 µm path length cell.

Representative data can be found in Fig. S11, with pertinent details in the caption.

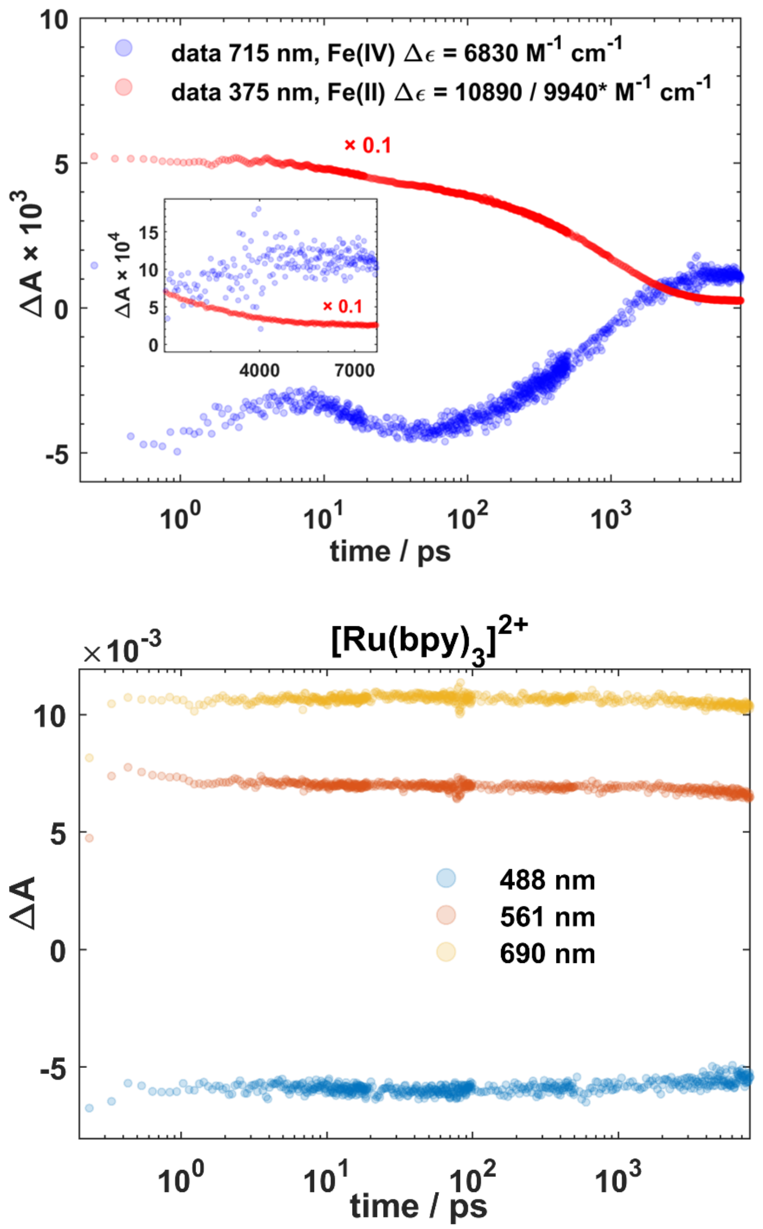

Figure S11: *Top:* Kinetics recorded at 375 nm (observed maximum of Fe(II)) and 715 nm (observed maximum of Fe(IV)). Note that the Fe(II) maximum is shifted from the product peak in the blue expected from spectroelectrochemistry (~355 nm). The first differential extinction coefficient mentioned for Fe(II) is for the maximum at 355 nm; the one which follows with the asterisk is for 375 nm. The obtained ΔA signals for the plateau at the end suggest final product concentrations of ca. 100 µM and 70 µM, from the Fe(II) and Fe(IV) peak, respectively. *Bottom:* Kinetic traces recorded at 488, 561 and 690 nm for $[Ru(bpy)_3]^{2+}$, in a 1000 µm path length cell. The obtained excited state concentrations are ca. 53, 75, 68 µM, which when scaled yield concentrations of 2120, 3000, and 2720 µM, respectively. The first value is neglected since 488 nm is close to the excitation wavelength, and the sample absorbance here was high resulting in low probe light transmission. Also, the maximum of the bleach at 448 nm could not be probed due to too much absorbance from the sample. At an excitation wavelength of 502 nm and the pulse energies employed (ca. 3 mJ/cm^2), contributions from $[Ru(bpy)_3]^{3+}$ and solvated electrons are negligible.[8] The total product yield can therefore be estimated to lie between 2−4%. Taken together with a quenching efficiency of ca. 0.65 from S2, the cage escape yield can be thought to lie between 3−6%.

5. Fitting Procedures

All data was corrected for group velocity dispersion of the probe light, i.e. “chirp” in Surface Explorer 4.2, which was also used to perform a preliminary single wavelength analysis (SWA) according to the following equation, where a sum of exponential decays was convolved with the instrument response function, IRF – assumed to be a Gaussian – whose parameters, t_0 (position) and the FWHM (full width at half maximum), Δ, were held fixed at 0.14 and 0.13 ps, respectively (determined from global analysis of the data, see below, where they were free-fit parameters; the latter, i.e. FWHM of the IRF was in good agreement with previous estimates made in our lab by fitting the Raman scatter in the solvent DMF in a 1 mm cuvette).

$S(t) = exp\left(-\frac{(t-t_0)^2}{{t_p}^2}\right) \otimes \sum_i A_i\, exp\left(-\frac{t-t_0}{\tau_i}\right);\quad here\ t_p = \frac{\Delta}{2\ ln\ 2},$ $\otimes$ is the convolution operator, A_i and τ_i are the amplitude and time constant of the exponential decay.

It was observed in the SWA that the rise component was sub-ps for blue wavelengths, while it got longer for wavelengths in the red (up to ca. 4 ps); this consequently effected the other component, making it shorter, which is an unavoidable situation when a sum of exponents is involved, since they are in fact correlated. At 625 nm, which is an isosbestic point in the excited state decay (Fig. S4) where any absorption changes are only due to formation and decay of the CS products, the ultrafast components obtained from a SWA were 2.4 and 10.4 ps, *i.e.* in reasonable agreement with the results of global analysis (main text, Scheme 1). The observed trend could also result from a wavelength dependent IRF, which was held fixed. In any case, the ultrafast component (and hence the subsequent decay component) can be expected to be highly sensitive to the IRF parameters, i.e. time-zero and shape, which necessarily complicates the analysis despite the use of convolution.

In light of this, the fs data was subject to global analysis using the software Glotaran[9,10] which serves as a graphical user interface for R-package “TIMP”. While Glotaran too performs a sum of exponentials convolved with the IRF fit as above, for an irreversible sequential decay in the simplest situation, the least squares regression is performed for all wavelengths simultaneously, so the obtained parameters can be thought to be relatively more robust, in the sense that they would present an average, if indeed a wavelength dependence is present in the data.

Global analysis of the data using a four-component fit model accounting for the IRF and coherent artifacts resulted in the time components and concentration profiles seen in Fig. S12. The coherent spectrum was found to resemble that of the excited state; if the data was fitted without accounting for a coherent artifact, then the fastest component was found to be sub-ps, ~0.54 ps, and the others were 21 and 913 ps. In both cases, the right singular vectors, which can be used to adjudge the goodness of the spectral fits, showed notable deviations in the blue, especially for the first component. This could potentially be due to the close resemblance of the spectral signature of the charge separated state and the excited state in the blue, where the only discernible difference could be regarded as a spectral blue-shift for the latter, moving the absorption peak from 375 nm to 360 nm in the initial timescales.

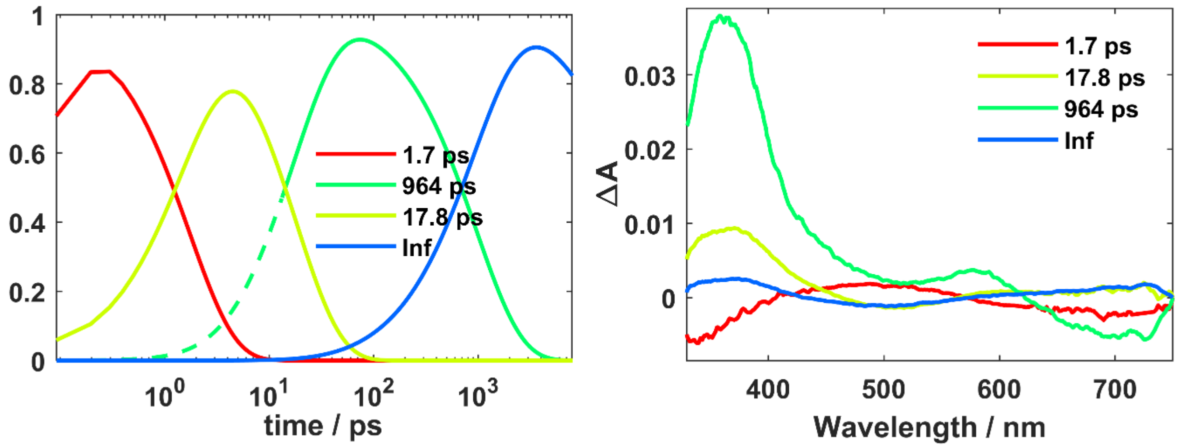

Figure S12: Time components obtained from global analysis of the data (left), plotted together with the DAS, decay associated spectra (right). Note the component associated with the excited state decay (aquamarine) is present from the beginning as a parallel pathway not correctly represented in this graph based on a sequential fit model.

Fits were also performed holding certain components fixed; e.g. the first component at 2 ps, or the second at 10 ps, and also both held fixed at 2 and 10 ps. This did not drastically impact fit quality, and the third component did not change by more than 2% in any case. Therefore, the numbers reported in the main text correspond to those obtained from an unconstrained fit with a coherent artifact. The DAS of the shortest time component (Fig. S12, right, red trace), <2 ps, mirrors the spectrum of the CS state very well, with the negative amplitudes in the blue and red showing the formation of Fe(II) and Fe(IV), respectively, and a reduction in the Fe(III) ground state bleach resulting in a positive band centred at ~500 nm. These geminate pairs undergo ultrafast recombination on a timescale of a few ten ps, also seen in the same figure, yellow trace. Diffusional pairs can be expected to react at later timescales, and the corresponding spectrum (aquamarine) associated with a time constant of close to ~1 ns matches well with that of the excited state, indicating that product formation, if present, is little, and cannot be resolved. The last spectrum recorded at 8 ns, and DAS with an infinite component, blue, are virtually identical to the product state spectrum.

6. ns-Transient Absorption Spectroscopy

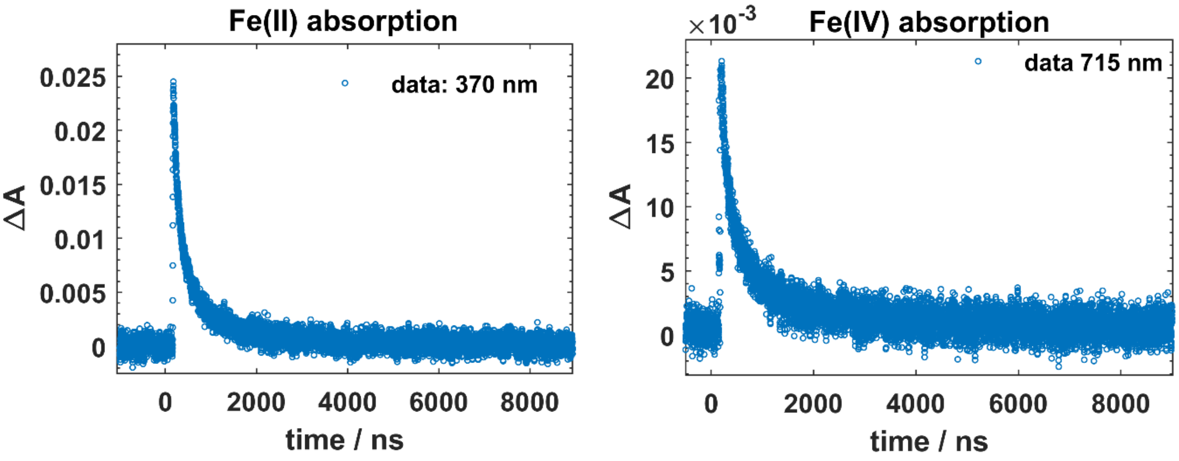

Figure S13: Kinetic traces at monitored at 370 nm (left) and 715 nm (right). $\lambda_{ex} = 465\ nm$, power = 15 mJ/pulse. The trace at 715 nm was corrected for fluorescence. Note that the Fe(II) will be competitively consumed by oxygen since the sample was sealed under air.

7. General Thermodynamic Considerations

The thermodynamic requirements for SB-CS in a transition metal complex as illustrated in Chart 1 c) of the main text for the case of an LMCT excited state are not specific to this type of excitation. The general design principle in form of an additional redox couple located in between the couples involved in the CT transition applies equally to complexes where the lowest excited CT state is of MLCT character as illustrated in Scheme S-1.

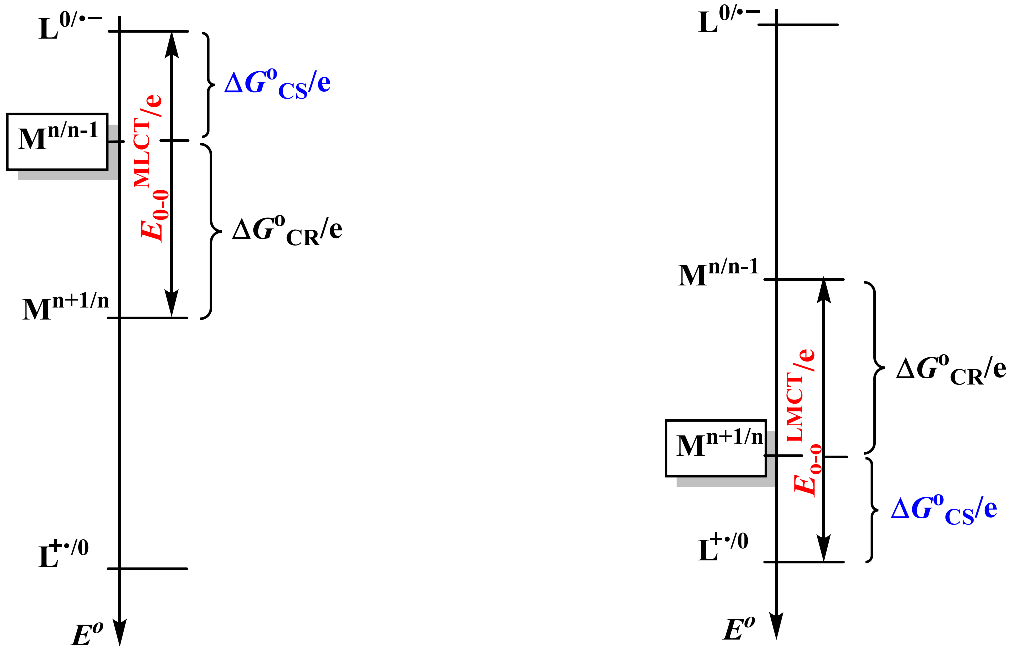

Scheme S-1. Thermodynamics of SB-CS for a TMC: Excited state energy ($E_{0\text{-}0}$) and free energies of charge separation (ΔG^o_{CS}) and recombination (ΔG^o_{CR}) as defined by the standard potentials (E^o) of the couples involved in MLCT excitation (left: $M^{n+1/n}$, $L^{0/\bullet-}$) or LMCT excitation (right: $M^{n/n-1}$, $L^{+\bullet/0}$) and an additional metal centered couple (boxed). In both cases the latter couple at intermediate potential ensures non-zero driving force for CS into M^{n-1} and M^{n+1} as long as $E_{0\text{-}0}$ can be approximated by the indicated potential difference.

8. References

(1) Materna, K. L.; Lalaoui, N.; Laureanti, J. A.; Walsh, A. P.; Rimgard, B. P.; Lomoth, R.; Thapper, A.; Ott, S.; Shaw, W. J.; Tian, H.; Hammarström, L. Using Surface Amide Couplings to Assemble Photocathodes for Solar Fuel Production Applications. *ACS Appl. Mater. Interfaces* **2020**, *12* (4), 4501–4509. https://doi.org/10.1021/acsami.9b19003.

(2) Van Turnhout, L.; Hattori, Y.; Meng, J.; Zheng, K.; Sá, J. Direct Observation of a Plasmon-Induced Hot Electron Flow in a Multimetallic Nanostructure. *Nano Lett.* **2020**, *20* (11), 8220–8228. https://doi.org/10.1021/acs.nanolett.0c03344.

(3) El-Zohry, A.; Orthaber, A.; Zietz, B. Isomerization and Aggregation of the Solar Cell Dye D149. *J. Phys. Chem. C* **2012**, *116* (50), 26144–26153. https://doi.org/10.1021/jp306636w.

(4) Lakowicz, J. R. *Principles of Fluorescence Spectroscopy*, 3rd ed.; Springer, 2006.

(5) Castanho, M. A. R. B.; Prieto, M. J. E. Fluorescence Quenching Data Interpretation in Biological Systems: The Use of Microscopic Models for Data Analysis and Interpretation of Complex Systems. *Biochim. Biophys. Acta - Biomembr.* **1998**, *1373* (1), 1–16. https://doi.org/https://doi.org/10.1016/S0005-2736(98)00081-9.

(6) Gehlen, M. H. The Centenary of the Stern-Volmer Equation of Fluorescence Quenching: From the Single Line Plot to the SV Quenching Map. *J. Photochem. Photobiol. C Photochem. Rev.* **2020**, *42*, 100338. https://doi.org/10.1016/j.jphotochemrev.2019.100338.

(7) Kjær, K. S.; Kaul, N.; Prakash, O.; Chábera, P.; Rosemann, N. W.; Honarfar, A.; Gordivska, O.; Fredin, L. A.; Bergquist, K. E.; Häggström, L.; Ericsson, T.; Lindh, L.; Yartsev, A.; Styring, S.; Huang, P.; Uhlig, J.; Bendix, J.; Strand, D.; Sundström, V.; Persson, P.; Lomoth, R.; Wärnmark, K. Luminescence and Reactivity of a Charge-Transfer Excited Iron Complex with Nanosecond Lifetime. *Science (80-.).* **2019**, *363* (6424), 249–253. https://doi.org/10.1126/science.aau7160.

(8) Muller, P.; Brettel, K. [Ru(Bpy)3]2+ as a Reference in Transient Absorption Spectroscopy: Differential Absorption Coefficients for Formation of the Long-Lived 3MLCT Excited State. *Photochem. Photobiol. Sci.* **2012**, *11*, 632–636. https://doi.org/10.1039/c2pp05333k.

(9) Snellenburg, J. J.; Laptenok, S.; Seger, R.; Mullen, K. M.; van Stokkum, I. H. M. Glotaran: A Java-Based Graphical User Interface for the R Package TIMP. *J. Stat. Software; Vol 1, Issue 3* **2012**.

(10) Van Stokkum, I. H. M.; Larsen, D. S.; Van Grondelle, R. Global and Target Analysis of Time-Resolved Spectra. *Biochim. Biophys. Acta - Bioenerg.* **2004**, *1657* (2–3), 82–104. https://doi.org/10.1016/j.bbabio.2004.04.011.
